# How orthodox protestant parents decide on the vaccination of their children: a qualitative study

**DOI:** 10.1186/1471-2458-12-408

**Published:** 2012-06-06

**Authors:** Wilhelmina L M Ruijs, Jeannine L A Hautvast, Giovanna van IJzendoorn, Wilke J C van Ansem, Koos van der Velden, Marlies EJL Hulscher

**Affiliations:** 1 Department of Primary and Community Care, Academic Collaborative Centre AMPHI, Radboud University Nijmegen Medical Centre, Geert Grooteplein 21, 6525 EZ, Nijmegen, The Netherlands; 2 Municipal Health Service GGD Rivierenland, J.S. de Jongplein 2, 4001 WG, Tiel, The Netherlands; 3 Scientific Institute for Quality of Healthcare, Radboud University Nijmegen Medical Centre, Geert Grooteplein 21, 6525EZ, Nijmegen, The Netherlands

**Keywords:** Decision-making, Immunization, Minority group, Religion, Religious objections to vaccination, Vaccination

## Abstract

**Background:**

Despite high vaccination coverage, there have recently been epidemics of vaccine preventable diseases in the Netherlands, largely confined to an orthodox protestant minority with religious objections to vaccination. The orthodox protestant minority consists of various denominations with either low, intermediate or high vaccination coverage. All orthodox protestant denominations leave the final decision to vaccinate or not up to their individual members.

**Methods:**

To gain insight into how orthodox protestant parents decide on vaccination, what arguments they use, and the consequences of their decisions, we conducted an in-depth interview study of both vaccinating and non-vaccinating orthodox protestant parents selected via purposeful sampling. The interviews were thematically coded by two analysts using the software program Atlas.ti. The initial coding results were reviewed, discussed, and refined by the analysts until consensus was reached. Emerging concepts were assessed for consistency using the constant comparative method from grounded theory.

**Results:**

After 27 interviews, data saturation was reached. Based on characteristics of the decision-making process (tradition vs. deliberation) and outcome (vaccinate or not), 4 subgroups of parents could be distinguished: traditionally non-vaccinating parents, deliberately non-vaccinating parents, deliberately vaccinating parents, and traditionally vaccinating parents. Except for the traditionally vaccinating parents, all used predominantly religious arguments to justify their vaccination decisions. Also with the exception of the traditionally vaccinating parents, all reported facing fears that they had made the wrong decision. This fear was most tangible among the deliberately vaccinating parents who thought they might be punished immediately by God for vaccinating their children and interpreted any side effects as a sign to stop vaccinating.

**Conclusions:**

Policy makers and health care professionals should stimulate orthodox protestant parents to make a deliberate vaccination choice but also realize that a deliberate choice does not necessarily mean a choice to vaccinate.

## Background

Despite high vaccination coverage, there have recently been measles, mumps, and rubella epidemics largely confined to an orthodox protestant minority that objects to vaccination in the Netherlands. [1-3] This orthodox protestant minority consists of about 250,000 individuals representing a number of denominations that separated from the Dutch Reformed Church. Each orthodox protestant denomination has its own specific interpretation of the confession, but predestination, election and the importance attached to intense, personal religious experiences play an important role in all of the denominations. Orthodox Protestants believe that God has predestined the fate of all human beings: only few are elected to live on in eternal bliss; they are informed of their blessed status by an intense religious experience.

Orthodox Protestants also constitute a cultural minority and have their own political party — the *Staatkundig Gereformeerde Partij* (SGP), their own newspaper, and their own schools. The social clustering of unvaccinated individuals promotes, however, the transmission of vaccine preventable diseases, and the measles, mumps and rubella epidemics even spread to their orthodox protestant relatives in Canada.

The orthodox protestant opposition to vaccination dates back to the nineteenth century. In 1823, the orthodox protestant physician Abraham Capadose published his objections to vaccination [4]. Referring to the severe side-effects of smallpox vaccination at that time, he stated that man was not allowed to cause disease in a healthy body. According to Capadose: Both health and disease were given by God and man should not interfere with divine providence. Although not all orthodox Protestants agreed with Capadose at the time, he nevertheless had many sympathizers. The introduction of compulsory smallpox vaccination for school entrance in 1872 and continuation of this to 1939 enhanced resistance to vaccination among orthodox Protestants [5].

In the 1960s, after the start of a National Immunization Program in the Netherlands, the incidence of the target diseases decreased sharply. However, outbreaks of vaccine preventable diseases confined to unvaccinated orthodox protestant minority groups still occurred. The polio epidemics of 1971, 1978, and 1992 led to particularly heated public debate because the general public could not understand the refusal to vaccinate young children who might otherwise be struck by this disabling disease [5-10]. Also among the orthodox Protestants, these polio epidemics fuelled a discussion of the acceptability of vaccination [11]. Biblical arguments in favor of vaccination were circulated by orthodox protestant opinion leaders [12]. And as a compromise, it was suggested that each congregation member was free to make his or her own personal decision and account for this to only God [13]. The final decision to vaccinate children or not is thus left to the orthodox protestant parents.

Parental decision making with regard to vaccination is a complex process. Not only religious considerations but also medical and psychosocial considerations can play a role [14-17]. Despite recurrent epidemics, there has been only one study — to our knowledge — of the motives to accept or refuse vaccination among orthodox Protestants. During the polio epidemic of 1978, Veenman and Jansma identified the following as major reasons for not being vaccinated prior to the outbreak of the epidemic: religious objections, family tradition, and fear of possible side-effects [18]. Many unvaccinated individuals subsequently changed their minds during this epidemic and decided to undergo vaccination afterall. Those who formerly objected to vaccination on religious grounds argued that, because the polio disease was so prevalent, vaccination did not constitute a preventive measure and was therefore allowed under these specific circumstances. In contrast, those who still refused vaccination viewed the epidemic as a test of their faith.

The aim of the present study in light of the societal circumstances outlined above is thus to gain insight into how orthodox protestant parents — without the immediate threat of an epidemic — decide to vaccinate or not vaccinate their children. The research questions were:

Do orthodox protestant parents make a deliberated decision with regard to the vaccination of their children?

What arguments do orthodox protestant parents use to justify their vaccination decisions?

What consequences of their decisions to vaccinate or not vaccinate do orthodox protestant parents face?

## Methods

### Research design

Because of the explorative character of or study we chose a qualitative research design and conducted in-depth, semi-structured interviews.

### Setting and study population

In the Netherlands, all children are offered a series of vaccinations free of charge by child health clinics under the auspices of the National Immunization Program. Vaccination is neither obligatory nor required for school entrance. The rate of voluntary vaccination is high: Vaccination coverage in the general population is about 95% [19]. Among the orthodox protestant minority, three subgroups can be distinguished on largely the basis of religious denomination: high coverage (>85%) for the Reformed Bond within the Protestant Church in the Netherlands and the Christian Reformed Churches; intermediate coverage (50–75%) for the Restored Reformed Church and the Reformed Congregations; and low coverage (<25%) for the Old Reformed Congregations and the Reformed Congregations in the Netherlands [20].

Our study population consisted of orthodox protestant parents who recently had to decide whether to vaccinate their young children or not. The study population was composed via purposeful sampling: vaccinating as well as non-vaccinating parents were recruited from various orthodox protestant denominations and various villages in the Dutch bible belt — an area of the Netherlands where orthodox protestants are concentrated (see below for further details). Inclusion in the study population was continued until thematic saturation was reached.

### Procedure

#### Recruitment

Participants were recruited via child health clinics in villages with low vaccination coverage due to religious objections. The selection of these villages was based on the results of a previous study [21]. We selected villages with low vaccination coverage and high numbers of orthodox protestants of a certain denomination, in order to include all denominations. We approached the local child health clinic professionals and asked them to select orthodox protestant parents who were willing to be interviewed. A snowball approach was also applied: Following the interviews, the participants were asked if they knew of other orthodox protestant parents — preferably from another denomination or another village — who might be willing to be interviewed as well. The intermediaries, namely the child health clinic professionals and interviewed parents, were given written information on the study to distribute to possible participants. When parents agreed to be interviewed, one of the researchers contacted them to explain the procedures further and answer any questions. An interview appointment was then made at a location, date, and time that was convenient to the parents.

#### Interview

The interviews were conducted in 2009 by trained interviewers (GvIJ and WLMR) with a medical background and no membership in one the orthodox protestant minority groups. Most interviews were conducted in home of the parents after obtaining informed consent. The interviewers used a list of topics that was based on information of key-informants such as orthodox protestant medical professionals, see Table 1. This topic list was loosely followed, starting with the composition of the family and vaccination status of the children. The interviews were of an exploratory nature and the interviewers did not express their opinions on vaccination or religion. At the end of the interview, the interviewees were explicitly asked if they had anything that had not yet been discussed to add. The average duration of the interviews was 60 minutes.

**Table 1:** Interview topics

	**Introduction**	
	Research on acceptance of vaccination among orthodox Protestants	
	Aim is to gain insight into the extent of vaccination and decision making with regard to such	
	**Main questions**	**Additional questions**
1	What is the composition of your family?	
2	Have you had your child/children vaccinated?	
	Why or why not?	Can you tell us more about this?
		Do other things play a role as well?
		-medical aspects
		-side effects
		- importance of having had childhood diseases
		- religious aspects
3	When did your decision making take place?	Before/during pregnancy?
		First months of life?
		Reconsideration with next child or in a new life phase ?
4	Who decides?	Roles of husband and wife.
		- Have you been vaccinated?
		- And your husband/wife?
		What does your family think about vaccination?
		- Has this influenced your decision?
		What do people in your church think about vaccination?
		- Has this influenced your decision? - Which church do you belong to?
5	Did you discuss your decision?	Asked for advice?
		- From whom?
6	Did you find it a difficult decision?	Have you ever regretted your decision?
		Did you previously think differently about vaccination?
7	For non-vaccinating:	
	What would you do during an epidemic?	Polio?
	What would do in case of an injury?	(Tetanus vaccination)
	What would you do when influenza vaccination is called for?	- Age
		- Medical grounds
	Specific circumstances : travel, work	(hepatitis B and influenza for nursing)
8	Do you talk about vaccination with your children?	Own opinions of older children?
		What would you think if your children later made a different decision?
9	What do you think of people who do/do not have their children vaccinated?	And if they belong to your own church?
10	Do you receive reactions to the fact that you are vaccinated/not vaccinated from your surroundings?	Do your surroundings know that you have been vaccinated/not been vaccinated?
		- Topic of conversation ?
		What kinds of reactions do you receive?
		- From whom?
11	For non-vaccinating:	
	How do doctors and other organizations react to your non-vaccination?	
12	Do you have anything that has not yet been addressed to add?	

#### Analysis

The interviews were recorded and transcribed verbatim. The transcripts were then analyzed thematically using the qualitative software program Atlas.ti. As our study had an explorative character we choose a grounded theory approach with an open coding sytem [22]. There were no predefined coding themes, the coding system was entirely based on the content of the data. Two analysts (WJCvA and WLMR) coded the transcripts independent of each other. The initial coding was reviewed, discussed, and refined until consensus could be achieved. Coding themes were for example “predestination” and “trust in God” that became both subcategories of “religious arguments”. All transcripts were coded and discussed by both analysts. The concepts emerging from the coding – such as the existence of four different subgroups of parents- were assessed using the constant comparative method from grounded theory. This means that when the concept of the four subgroups was identified, previously analyzed interviews were reviewed in order to check if their content fitted into this concept.

#### Ethics

The study was approved by the research ethics committee of the Radboud University Nijmegen Medical Centre, Nijmegen, The Netherlands; CMO number 2010/462.

## Results

### Participant characteristics

Initially 28 orthodox protestant families were approached, one family did not participate because of practical constraints. From 27 families, we interviewed one or both parents: 21 mothers, 3 fathers, and 3 couples. The families belonged to various denominations and 13 families started vaccinating their children. Further details are shown in Table 2.

**Table 2:** Characteristics of orthodox protestant parents participating in study

	Total	Traditionally NON-vaccinating parents	Deliberately NON-vaccinating parents	Deliberately vaccinating parents^4^	Traditionally vaccinating parents
*Participating families*	27	8	6	9	4
***Interviewee(s)***					
Mother	21	7	4	8	2
Father	3	1	1	0	1
Both parents	3	0	1	1	1
***Vaccination coverage for denomination***[20]					
High^1^	3	0	1	2	0
Intermediate^2^	14	0	4	6	4
Low^3^	10	8	1	1	0
***Number of children***					
1-2	11	2	2	5	2
3-4	8	3	2	1	2
5-11	8	3	2	3	0

^1^ Protestant Church in the Netherlands, Reformed Bond, and Christian Reformed Churches.

^2^ Restored Reformed Church and Reformed Congregations.

^3^ Reformed Congregations in the Netherlands and Old Reformed Congregations.

^4^ Two families stopped vaccination because of the occurrence of unexpected medical events; they are nevertheless included here.

### The decision-making process: tradition versus deliberate choice

The majority of parents decided around the birth of their first child on whether or not they would take part in the National Immunization Program. With regard to the vaccination decision-making process, two subgroups of parents could be distinguished: parents who followed tradition versus parents who made a deliberate choice.

The parents who followed tradition did not go through an explicit decision-making process. They hardly discussed the topic of vaccination and simply did the same as their parents. If they came from a non-vaccinating family, they refused vaccination; if they came from a vaccinating family, they agreed to vaccination.

"“We were both a member of the same type of congregation; that makes difference. You have been given the same values. It was no longer a point of discussion.” (Respondent 5, traditionally non-vaccinating family)"

"“Yes, did we really think about it? We didn’t really consciously think about it because both of us have also been vaccinated. You just continue on, really … I wouldn’t know of anyone in my family who hasn’t done it.”(Respondent 11, traditionally vaccinating family)"

Those parents who made a deliberate choice considered both to vaccinate and not to vaccinate. Although the man is the head of the family in orthodox Protestantism, in the cases in our study of making a deliberate decision, the decision was mostly made by the two parents after lengthy discussion. Some of the couples making a deliberate choice first discussed the topic with their parents or asked their friends’ opinion. None of the participants making a deliberate choice discussed the topic with the religious leaders of their churches. Personal religious experiences were sometimes reported to play an important role in their final decisions, however. Many of the parents making a deliberate choice prayed to God to help them with their decision and some reported having received a sign from God.

"“… I thus put my bible down on the seat of the car and, just before I got to the Public Health Building, I opened up the bible and there it stood, that the stuff that is given may be used. Things were clear for me then.” (Respondent 13)"

For both parents who followed tradition and parents who made a deliberate choice, the vaccination decision was made for all children to come. Although some parents reported reconsidering the decision with the birth of every new child, this did not lead to a different decision. Moreover, all of the parents agreed that the parents are responsible for the vaccination decisions as long as the children live in their homes; the children take on responsibility when they come of age and marry.

### The final decision: four subgroups of parents and their arguments

When the nature of the vaccination decision-making process is considered together with the final outcome regarding participation in the National Immunization Program (i.e., vaccination) or not, four subgroups of orthodox protestant parents could be distinguished:

1) parents who followed tradition and refused vaccination,

2) parents who made a deliberate choice and decided against vaccination,

3) parents who made a deliberate choice and decided in favor of vaccination, and

4) parents who followed tradition and agreed to vaccination.

The characteristics of the respondents in each subgroup are summarized in Table 2.

The subgroups are described in more detail below.

#### Traditionally non-vaccinating parents

The traditionally non-vaccinating parents all belonged to denominations with low vaccination coverage. They referred to religious doctrine to explain their refusal of vaccination. Man should not interfere with divine providence and man *cannot* interfere with divine providence because God is almighty. The timing of a medical intervention is of critical importance for them: Preventive measures are not accepted while curative and palliative measures often are.

"“Whether I have my children vaccinated or not does not matter to me because I don’t believe in it. I believe that if God wants to spare my children from an accident, then He will spare them from it.” (Respondent 1)"

"“This is even strengthened by all that I have been through….You can simply see that you have nothing to say.” (Respondent 26)"

"“Because we believe that there is a God who steers our lives and leads us and that we should not get ahead of his deeds. We cannot predict what he brings or does not bring upon us.” (Respondent 16)"

Tetanus post-exposure prophylaxis was typically considered a cure and thus accepted by these parents. Some of the traditional non-vaccinating parents in our study therefore also accepted polio vaccination in the case of an epidemic. When faced with immediate danger, vaccination was no longer considered preventive by them.

"“I can remember when polio was rampant; you could be given a sugar cube with the virus, that is what they recommended and many of us — including myself — swallowed such a cube. But there was a real danger then. And that’s something different, in my opinion.”(Respondent 24)"

Apart from their religious objections, the traditionally non-vaccinating parents sometimes had concerns about vaccine safety and particularly about the disease-inducing properties of vaccines, however they reported these concerns were not decisive. They were still used to the presence of infectious childhood diseases like mumps and measles, which they did not consider very serious.

"“You don’t have any complaint or any disease. And then you inject something that makes your child sick.” (Respondent 16)"

"“But a childhood disease…to immunize against it? Looking at the children, they simply come down with it. I also had it earlier myself. And you get over it; it’s just part of things.” (Respondent 9)"

#### Deliberately non-vaccinating parents

Deliberately non-vaccinating parents often live in a community with both vaccinating and non-vaccinating orthodox Protestants, for example, one of the spouses has been vaccinated while the other has not. These parents also used predominantly religious arguments but mostly in connection with their trust in God. Even if God sends a disease, he has a purpose for it. The personal relationship with God plays a major role in the decision to not vaccinate; the parents put all their trust in God. Such experiences as life-threatening diseases only enhance one’s relationship with God. Deliberately non-vaccinating parents stress the significance of the disease rather than deny the medical effectiveness of vaccination.

"“I know for sure that God cares for me. And that the things He sends me, that may also be disease, that He will help me to cope with it.” (Respondent 23)"

"“I mean, I say to myself afterwards — I hope that I never have to go through this again — but it has been really good for our family, our marriage, but also our religious life. Through this we live closer to God.” (Respondent 10)"

"“And purely without looking at the bible, I have to say that it looks like the vaccination program has had paid off as far as the immunization goes.”(Respondent 23)"

In contrast to the other deliberately non-vaccinating parents, one orthodox protestant couple – both from a traditionally vaccinating background- decided against vaccination of their children for non-religious reasons; they were convinced that vaccines could have major side-effects and therefore preferred their children to acquire immunity by conquering infections with the aid of homeopathy.

#### Deliberately vaccinating parents

The deliberately vaccinating parents were mostly *not* vaccinated themselves. After lengthy discussions, they decided to break with a longstanding tradition in their families.Although they cite the medical benefits of vaccination, they used predominantly religious arguments to justify their decision to vaccinate. They consider vaccination a gift from God to be used in gratitude. However, in the interviews, they elaborated more on the counterarguments to the religious objections to vaccination than on their own arguments in favor of vaccination. These parents reported that, after thinking things over, they could not see any good reason to *not* vaccinate.

"“Yes, you may use the means that are there and I am convinced that it says in the bible that the Lord Jesus himself also says at a given point that … you have flat roofs in Israel, and then he says that fences should be put around them because otherwise they fall off.” (Respondent 7)"

"“For me, the Lord is not bound to vaccination. Then I would think of God in much too little terms. If he was bound to vaccination. If he really wants something to happen to us, then he is not dependent on vaccination.”(Respondent 13)"

"“I simply lack the faith; I don’t have it. When you hear some stories or read some books, they have such a faith…But that faith, I don’t have it.” (Respondent 22)"

"“Because you want to protect your children against everything…” (Respondent 8)"

#### Traditionally vaccinating parents

Traditionally vaccinating parents were vaccinated themselves and did not see any religious objections to vaccination. They did not relate the issue of vaccination to their belief in God. Medical arguments were used to justify their decision. If they had any doubts about vaccination, these concerned the possible adverse effects of the immunization itself.

"“I cannot say that I know someone who does not do it. I have the idea that by us in the church, certainly here, that it’s simply accepted….I also cannot think up any arguments for why it should not be allowed.”(Respondent 15)"

"“I have also thus seen that you should not underestimate these illnesses….but I think then, well, what does it do with the immune system of your child?”(Respondent 9)"

### Psychosocial consequences

Many orthodox protestant parents feared to regret their decision on vaccination in future. The traditionally and deliberately non-vaccinating parents both considered epidemics — and particularly polio epidemics — to be an ordeal and feared that their faith would not be sufficiently strong to endure it. But most of all, they feared their children possibly becoming severely ill and dying.

"[In case of a polio epidemic] I think that I would end up in a real dip. The struggle then begins. Maybe I should have [vaccinated them]; then they would have maybe [not have become ill]…(Respondent 1, traditionally non-vaccinating family)"

"[In case of a polio epidemic] I would really find it horrible if one of my children or my husband would get it, I really would. I cannot bear to think of it. And I count on being spared of this. I would try to explain later to my child why I didn’t do it, purely on the basis of faith. (Respondent 10, deliberately non-vaccinating family)"

On the other hand “first generation” deliberately vaccinating parents feared the adverse effects of vaccination as these are taken as a sign from God that they have made the wrong decision. Two deliberately vaccinating parents, for example, stopped the vaccination series when unexpected medical events arose. In one case, the daughter still came down with the measles after being vaccinated. In the other case, serious adverse effects arose but were later found to be the symptoms of an underlying disease. In light of apparent adverse vaccination effects, the mother did not dare to continue vaccination. In her opinion and in response to her prayers, she had received a sign from God to stop vaccination.

"“Imagine that the decision is wrong. Just a bit of fear, because you made a decision on rational grounds but more than just the rational may be at play. You read, of course, about the possible effects and, certainly when I first had her vaccinated, I found it scary. You break with something you grew up with.”(Respondent 21)"

"“And I was really shocked by that… I didn’t dare to talk with anyone about it simply because I, myself, thought that I had done it. I found the guilt on my part to be so heavy…, that I really didn’t talk to anyone about it…(Respondent 4)"

"Now, yeah, I wanted to know for sure for myself whether I could continue or not. I didn’t know for myself but also didn’t dare to anymore[…] and then I prayed specifically: “Lord, if you want us to no longer vaccinate, then let the oldest who has had all the vaccinations get the mumps. Now, a couple of weeks later, he came down with the mumps. I was certain about things then. (Respondent 4)"

Referring to the generally very high vaccination coverage in The Netherlands, some non-vaccinating parents reported discussions with colleagues or neighbors who did not understand their objections to vaccination. On the other hand, some of the deliberately vaccinating parents — particularly those living in a largely non-vaccinating community — mentioned feeling uncomfortable in light of social control. They did not dare to speak of their decision to vaccinate with members of the congregation or even family members.

"“Because if there’s the mumps or the measles, that’s the talk of the day at school and they ask out of interest if we have already had them. I don’t tell them that we’ve been vaccinated then but simply say nothing. I just walk a bit further up if I notice that they’re talking about it.” (Respondent 22, deliberately vaccinating family)"

Only the traditionally vaccinating parents did not report any psychosocial consequences of their decision to vaccinate.

## Discussion

In terms of the process underlying the decision to vaccinate or not vaccinate, the orthodox protestant parents in our study could be divided into those who were guided by tradition and those who made a deliberate choice. In combination with the actual decision, this produced four subgroups: traditionally non-vaccinating parents, deliberately non-vaccinating parents, deliberately vaccinating parents, and traditionally vaccinating parents. All subgroups –except the traditionally vaccinating parents- used predominantly religious arguments to justify their decision. And all subgroups –except the traditionally vaccinating parents- reported psychosocial consequences of their decision.

### Tradition versus deliberate choice

Many of the orthodox protestant parents in our study reported simply following the tradition in their families. Tradition is indeed an important factor in the acceptance or refusal of vaccination — not only among orthodox protestants [14]. “Band wagoning” or going along with the majority was first described in connection with vaccination decision-making in 1994 [15]. In the general population, band wagoning plays an important role as the *majority* of vaccination decisions in the Netherlands are made without much deliberation [23]. For traditionally non-vaccinating orthodox protestant parents, refusing vaccination is part of a longstanding tradition and therefore part of the group’s identity [9]. Moreover, in orthodox Protestantism following tradition is valued and thus has a positive connotation [24]. During the interviews, some traditionally non-vaccinating respondents referred to the biblical tribe of the Rechabites who were known for their fidelity to the customs of their ancestors. Veenman and Jansma also reported tradition to play an important role in the vaccination decision-making among the most conservative orthodox protestant denominations. This was attributed to the paucity of contact with vaccinating individuals within these denominations at that time [18].

For the parents who made a deliberate choice on vaccination, the trigger for thinking things over was most often the birth of their first child. The same was found in a study of vaccination decision-making among orthodox protestant families in the Dutch province of Zuid-Holland [25]. Interventions aimed at stimulating deliberate decision-making, instead of following tradition, should therefore focus on the parents of firstborns.

### Religious versus medical arguments

Three of the four subgroups distinguished in this study offered predominantly religious arguments to justify their vaccination decisions. Medical arguments thus appeared to be of minor importance among orthodox protestant parents.

These findings are in line with the results of previous research showing that orthodox protestant youngsters in the Netherlands were far more interested in the religious aspects of vaccination than in the medical aspects [26]. In a Canadian study on refusal of immunization, it was also reported that for Dutch immigrants (belonging to religious congregations related to the denominations described here) religious arguments were decisive [27]. However, in both these studies orthodox Protestants who accepted vaccination were not included. Our finding that “first generation” deliberately vaccinating parents also predominantly use religious arguments indicates that non-vaccinating orthodox protestant parents will probably not be convinced by medical arguments to change their position towards vaccination.

In our study the subgroup of traditionally vaccinating parents was the only subgroup of parents that offered predominantly medical arguments to justify their choice. Like among other –not orthodox protestant– parents in the Netherlands some of them had doubts on the safety of vaccines [23], these doubts, however, did not (yet) result in refusal of vaccination. In a systematic review of qualitative studies on parental attitude towards vaccination, ‘concern on the safety of vaccines’ was the most reported barrier to vaccination [28]. Although this concern was also reported by some traditionally non-vaccinating parents, it was not decisive for them. Moreover, for the traditionally non-vaccinating parents lack of vaccine safety had a religious connotation: Man is not allowed to cause disease in a by God given healthy body [4,13]. Therefore, regarding their arguments, the deliberately non-vaccinating family who did not vaccinate their children because of doubts on vaccine safety fits better in the general population in the Netherlands than in the non-vaccinating orthodox protestant subgroups described above.

### Psychosocial consequences

For all of the orthodox protestant parents in our study with the exception of the traditional vaccinating parents, the vaccination decision was accompanied by a considerable fear of the consequences. This fear was most tangible among the deliberately vaccinating parents who feared immediate punishment. For parents in doubt, this fear may be a reason to refrain from vaccination — also because errors of omission (and thus not vaccinating) are generally “preferred” over errors of commission [16]. For parents facing adverse effects of vaccination, this fear may be the reason to stop vaccinating as found in the present study.

### Other factors possibly influencing acceptance of vaccination

#### *Trust in the provider*

In qualitative studies on acceptance of vaccination, trust in the provider of childhood vaccinations and the medical community in general is identified as an important and possibly decisive factor [17,28]. For the orthodox protestant parents we interviewed, this trust -or lack of trust- in the provider seemed, however, not an issue. Like almost all parents in the Netherlands they regularly visited the child health clinics, if not for vaccination then for monitoring growth and development. Moreover, for the general population in The Netherlands, lack of trust in the provider of childhood vaccinations seems not a major issue either [29].

#### *Socio-economic factors*

In the international literature, socio-economic factors are often mentioned as an explanation for low vaccination coverage. One possible reason for refraining from vaccination may indeed be a lack of insurance [30,31]. In the Netherlands, however, vaccination via the National Immunization Program is provided by the government, free of charge. Although some orthodox protestant parents are uninsured because they think that insurance interferes with divine providence, the costs cannot be the reason for refraining from vaccination. Moreover, the group of uninsured orthodox protestants is only about 11,000 and is thus considerably smaller than the group refusing vaccination [32].

#### *Position of women*

Another issue possibly influencing vaccination coverage is the position of women within the orthodox protestant minority. Until 2006, the orthodox protestant political party (SGP) did not accept female members because “the man is the head of the woman” and married women are expected to stay at home to care for the children [9]. Particularly in the most conservative denominations, education is considered less important for girls than for boys [33]. Given that maternal educational level is an important determinant of child health [34], the position of women in a religious minority might influence vaccination coverage as well. In the orthodox protestant minority in the Netherlands, the educational level of the mother indeed correlates positively with the child being vaccinated [35]. While few orthodox protestant girls enter university, they now have the same representation as other Dutch girls in the different levels of secondary education in the Netherlands and even outnumber orthodox protestant boys in the higher levels of secondary education [36]. This increase in educational level among orthodox protestant females may thus lead to increased acceptance of vaccination, by deliberately vaccinating parents in future.

### Strengths and limitations

#### Generalizability

This study focuses on a specific religious minority in The Netherlands. Detailed information on their decision-making on vaccination is important for public health policy in The Netherlands. The generalizability of our results to religious minorities with low vaccination coverage in other countries is, however, limited. Among the orthodox protestants we described, objections to vaccination are rooted in the religion itself. In other religious minorities with low vaccination coverage, there may be other barriers to vaccination, such as practical constraints or complot theories. [37-40] Nevertheless, it is important to keep in mind that religious minorities with objections to vaccination will probably not be convinced to change their position by medical arguments.

#### Recruitment of participants

The orthodox protestants in the Netherlands are a hard-to-reach minority [20]. Therefore we recruited our participants via intermediaries and a snowball method. Especially the snowball method may lead to overrepresentation of subgroups that are already enrolled. In order to ensure that all orthodox protestant subgroups were represented we specifically sought vaccinating as well as non-vaccinating parents of denominations not yet (sufficiently) included. Moreover we continued inclusion until data saturation was reached.

In the traditional orthodox protestant role pattern the woman cares for the children and visits the child healthcare centre with them. Our recruitment methods thus resulted in an overrepresentation of women. Although according to orthodox protestant customs the man is the head of the family, we do not consider this a problem. Regarding vaccination the woman is expected to carry out the couples decision, and she is trusted by her husband to do so.

#### Social desirability

As for orthodox protestants vaccination is a delicate subject, we chose semi-structured interviews as method to explore the decision-making. However, interviews are by definition subjective and prone to social desirability bias. In order to prevent social desirable answers the interviewers tried to create a confidential atmosphere. They were respectful regarding the religious beliefs of the participants and did not express their opinions on vaccination. Because of the private nature of the decision-making, triangulation was not feasible. Nevertheless we think we have sufficiently combated social desirability bias by including a vaccinating parent belonging to a denomination with low vaccination coverage as well as non-vaccinating parents belonging to denominations with high vaccination coverage.

## Conclusions

Based on the decision-making process (i.e., follow tradition or make a deliberate choice) and the outcome (i.e., vaccinate or not), four subgroups of orthodox protestant parents could be distinguished: traditional non-vaccinating parents, deliberately non-vaccinating parents, deliberately vaccinating parents, and traditional vaccinating parents. All of the subgroups with the exception of the traditional vaccinating parents offered predominantly religious arguments to justify their vaccination decision. Similarly, all of the subgroups with the exception of the traditional vaccinating parents faced fears that they had made the wrong choice.

Policymakers and health care professionals can play an important role in stimulating orthodox protestant parents to make a deliberate choice on vaccination. In doing this, however, they should realize that a deliberate choice does not necessarily mean a choice in favor of vaccination. Moreover, they can play an active role in handling the consequences of a particular decision by informing vaccinating parents of adverse vaccination effects and how to deal with them, and giving non-vaccinating parents a second chance for vaccination. Although health is an important value, the vaccination decision making of orthodox protestant parents shows health to not be the only important value in life — at least for them.

## Competing interests

The authors declare that they have no competing interests.

## Authors’ contributions

WLMR conceived of the study, participated in the design, collected data, participated in analyses and drafted the manuscript. JLAH participated in the design of the study and helped to draft the manuscript. GvIJ collected data and revised the manuscript, WJCvA participated in analyses and revised the manuscript, KvdV participated in the design of the study and revised the manuscript. MEJL participated in the design of the study and helped to draft the manuscript. All authors read and approved the final manuscript.

## Pre-publication history

The pre-publication history for this paper can be accessed here:

http://www.biomedcentral.com/1471-2458/12/408/prepub
